# Chemical Pneumonitis and Reactive Airways Dysfunction Syndrome After Fumigation Exposure to Dimethanol and Glutaraldehyde: A Silent Menace Unmasked

**DOI:** 10.7759/cureus.42939

**Published:** 2023-08-04

**Authors:** Keyur Saboo, Rinkle R Gemnani, Soumya Sharma, Sunil Kumar, Sourya Acharya

**Affiliations:** 1 Medicine, Jawaharlal Nehru Medical College, Wardha, IND; 2 Ophthalmology, Jawaharlal Nehru Medical College, Wardha, IND

**Keywords:** case report, acute respiratory distress syndrome, health professionals, fumigation, chemical pneumonitis

## Abstract

Chemical pneumonitis caused by dimethanol and glutaraldehyde exposure is a serious medical condition that requires prompt and effective treatment. As per a literature search in Google Scholar, PubMed, and Scopus, this is the first instance of chemical pneumonitis caused after fumigation with dimethanol and glutaraldehyde inhalation. This article discusses the factors that can contribute to the development of chemical pneumonitis and outlines the diagnostic and treatment options available to healthcare professionals. By understanding the causes and consequences of dimethanol- and glutaraldehyde-induced chemical pneumonitis, medical professionals can provide better care to their patients and help prevent future cases of this potentially life-threatening condition. This describes a case of a 60-year-old female who presented to the emergency department complaining of acute onset of shortness of breath approximately 48 hours after being exposed to dimethanol and glutaraldehyde while working in intensive care. After 13 days, the patient’s symptoms subsided and she was discharged. On follow-up, after 1 month, there was a marked resolution of the initial symptoms.

## Introduction

Dimethanol and glutaraldehyde are common solvents used in various industries for their ability to dissolve a wide range of substances. While they have many practical applications, exposure to these chemicals can be harmful and even life-threatening. One potential complication of dimethanol and glutaraldehyde exposure is chemical pneumonitis, a condition characterized by inflammation of the lungs. These chemicals are utilized in industrial, laboratory, agricultural, medical, and some domestic settings, particularly for sterilizing and cleaning surfaces and equipment.

Glutaraldehyde and dimethanol are colorless, oily liquids with a harsh, unpleasant smell [1]. Patients who already have atopy or pulmonary issues are more likely to experience adverse respiratory events, which typically occur after extended exposure to these products. According to animal studies, severe necrotizing pneumonia is the main pathological alteration. Other findings include damage to alveolar septae, capillaries in the pulmonary system, and severe damage to the airway epithelium, along with disintegration of the lipid surfactant layer. Additional pathological signs include atelectasis, interstitial inflammation, and the development of hyaline membranes [2,3].

Chemical pneumonitis is a type of acute lung injury caused by inhaling or aspirating irritant substances, including gases, vapors, fumes, or particulates. Dimethanol and glutaraldehyde are colorless, odorless solvents commonly used in various industries, such as paint, varnish, and adhesive manufacturing. These chemicals are known to be toxic to the respiratory system, especially when exposed to high concentrations or for prolonged periods [4,5]. However, we present a case of a patient with no prior history of illness, who experienced serious side effects after just one exposure to a fumigation product.

## Case presentation

A 60-year-old woman with a cough, breathing problems, and chest pain was taken to the emergency room (ER) at five in the morning. She was accidentally exposed to glutaraldehyde and dimethanol while working in an Intensive Care Unit (ICU) setup involving the same solvent. Initially, the woman experienced minor symptoms, including a sore throat after exposure, for which she sought treatment at a nearby hospital. However, despite taking medication at home for two days, her symptoms worsened, leading to her subsequent visit to our emergency room.

The patient was in distress and had a 70% oxygen saturation on room air when she arrived at the emergency department. Her vital signs were as follows: blood pressure of 118/78 mmHg, heart rate of 110 beats per minute, respiratory rate of 24 breaths per minute, and temperature of 38.2°C. On physical examination, she appeared anxious and uncomfortable, with bilateral coarse crackles and wheezes on lung auscultation.

**Figure 1:**
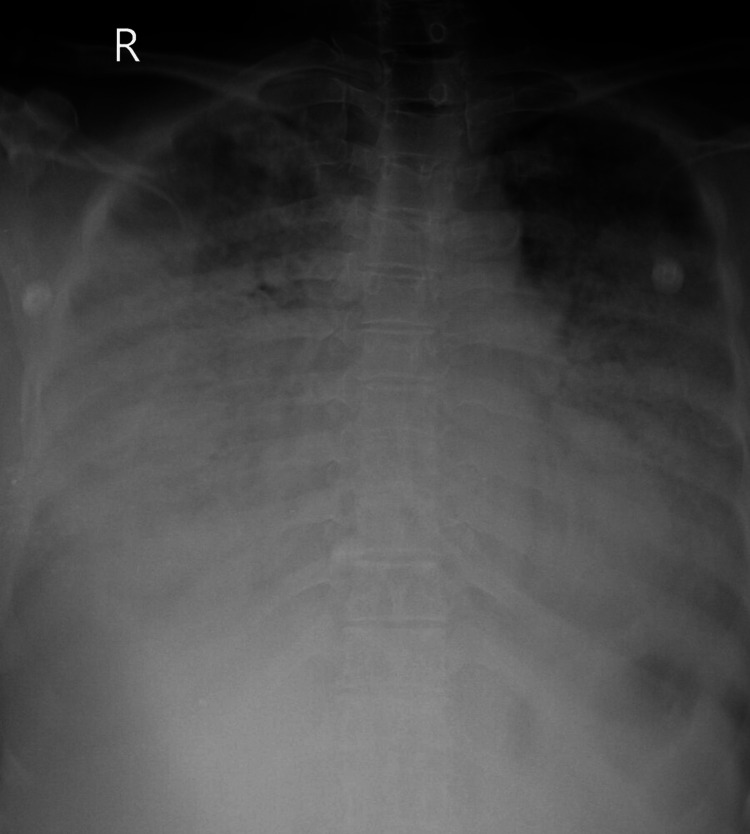
Chest X-ray on admission suggestive of bilateral extensive haziness (white-out lung).

**Figure 2:**
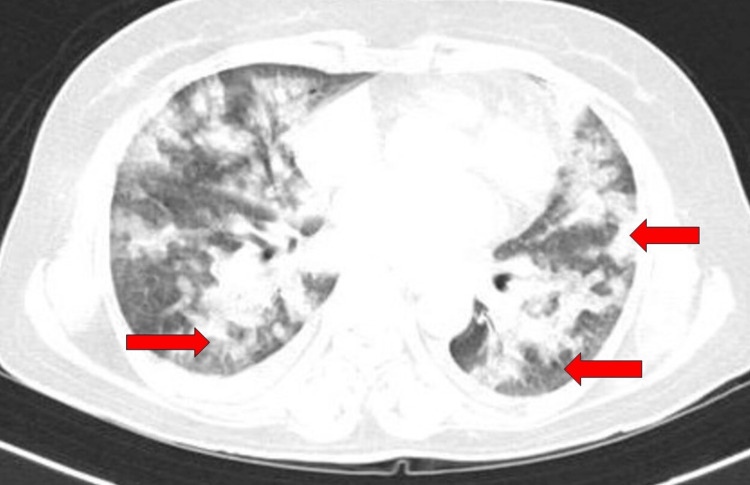
HRCT thorax suggestive of extensive ground glass opacities in the upper and lower lobes of both lungs (red arrow). HRCT: high-resolution computed tomography.

Based on her clinical presentation and history, we suspected chemical pneumonitis caused by dimethanol and glutaraldehyde exposure. A chest x-ray was taken (Figure 1), revealing diffuse bilateral infiltrates suggestive of acute lung injury. We initiated supplemental oxygen via a face mask, nebulized bronchodilators, and intravenous corticosteroids. Her arterial blood gas readings on room air were as follows: pH 7.42, pCO2 43.6 mmHg, pO2 56.2 mmHg, and bicarbonate (HCO_3_) 24.3 mmol/L. Normal blood gas measurements are typically between 7.35 and 7.45 for pH, 80 to 100 mmHg for pO2, 36 to 45 mmHg for pCO2, and 22 to 26 mmol/L for bicarbonate (HCO_3_) levels. With the exception of a white blood cell count of 16.9 k/mm^3^ (normal range: 4.5 to 11.0 k/mm^3^), the high-resolution computed tomography (HRCT) of the thorax showed extensive ground glass opacities in the upper and lower lobes of both lungs (Figure 2). Viral respiratory pathogen testing using polymerase chain reaction (PCR) returned negative results, and respiratory cultures showed no growth of pathogenic microbes. There were increased levels of inflammatory indicators such as C-reactive protein, D-dimer, ferritin, lactate dehydrogenase, and erythrocyte sedimentation rate (ESR). All other laboratory findings fell within acceptable limits (Table 1). The electrocardiogram showed a normal sinus rhythm with a ventricular rate of 98 beats per minute, a normal axis, and normal intervals. The 2D echocardiography indicated normal findings, with a left ventricular ejection fraction of 60%.

**Table 1:** Investigation profile of the patient

Investigations	Patient Values	Reference Values
Hemoglobin	11.2 g/dl	13-17 g/dl
Total leukocyte count	24,600 /dl	4000-11,000/dl
Platelet count	364,000/dl	150,000-400,000/dl
Serum creatinine	0.9 mg/dl	0.5-1.2 mg/dl
Albumin	3.2 g/dl	3.5-5.0 g/dl
Aspartate aminotransferase	534 U/l	<50 U/l
Alanine aminotransferase	344 U/l	17-59 U/l
Total bilirubin	1.6 mg/dl	0.2-1.3 mg/dl
C-reactive protein	64 mg/dl	<6 mg/dl
D-dIMER	988 ng/ml	<500 ng/ml
Erythrocyte sedimentation rate	82 mm/hr	0-20 mm/hr
Ferritin	755 ng/ml	11.1-264 ng/ml

**Figure 3:**
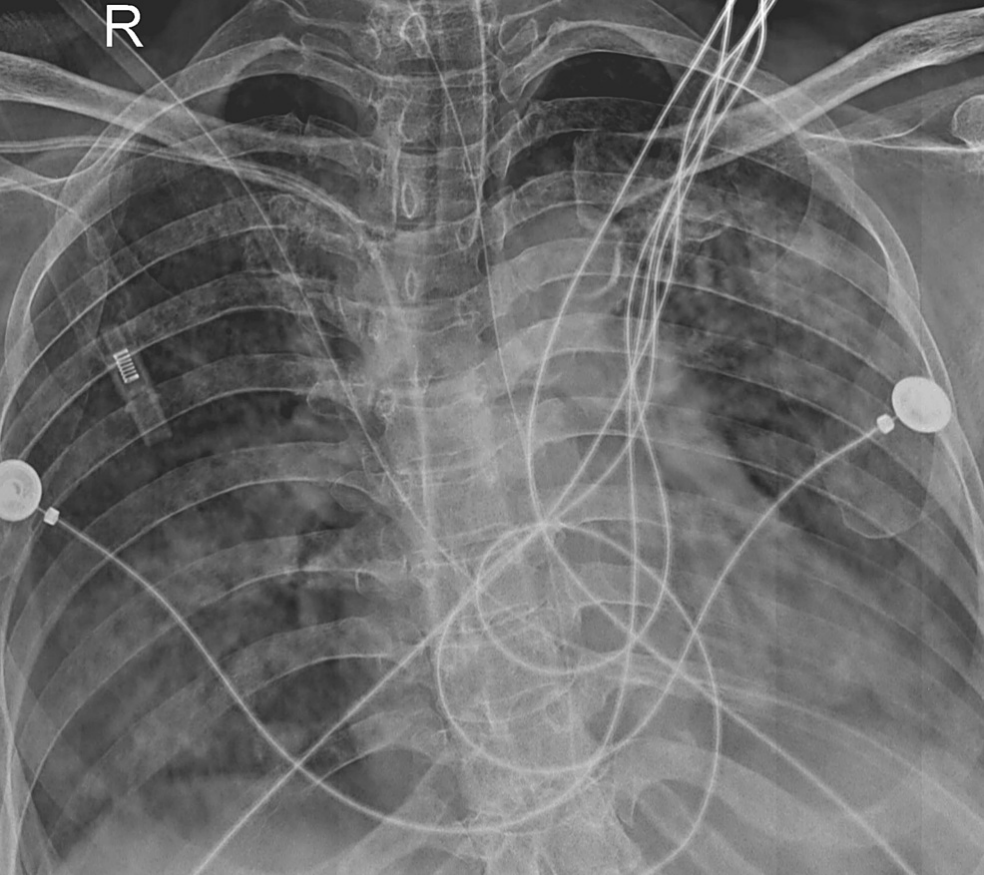
Chest X-ray on Day 6 of admission showed resolution.

**Figure 4:**
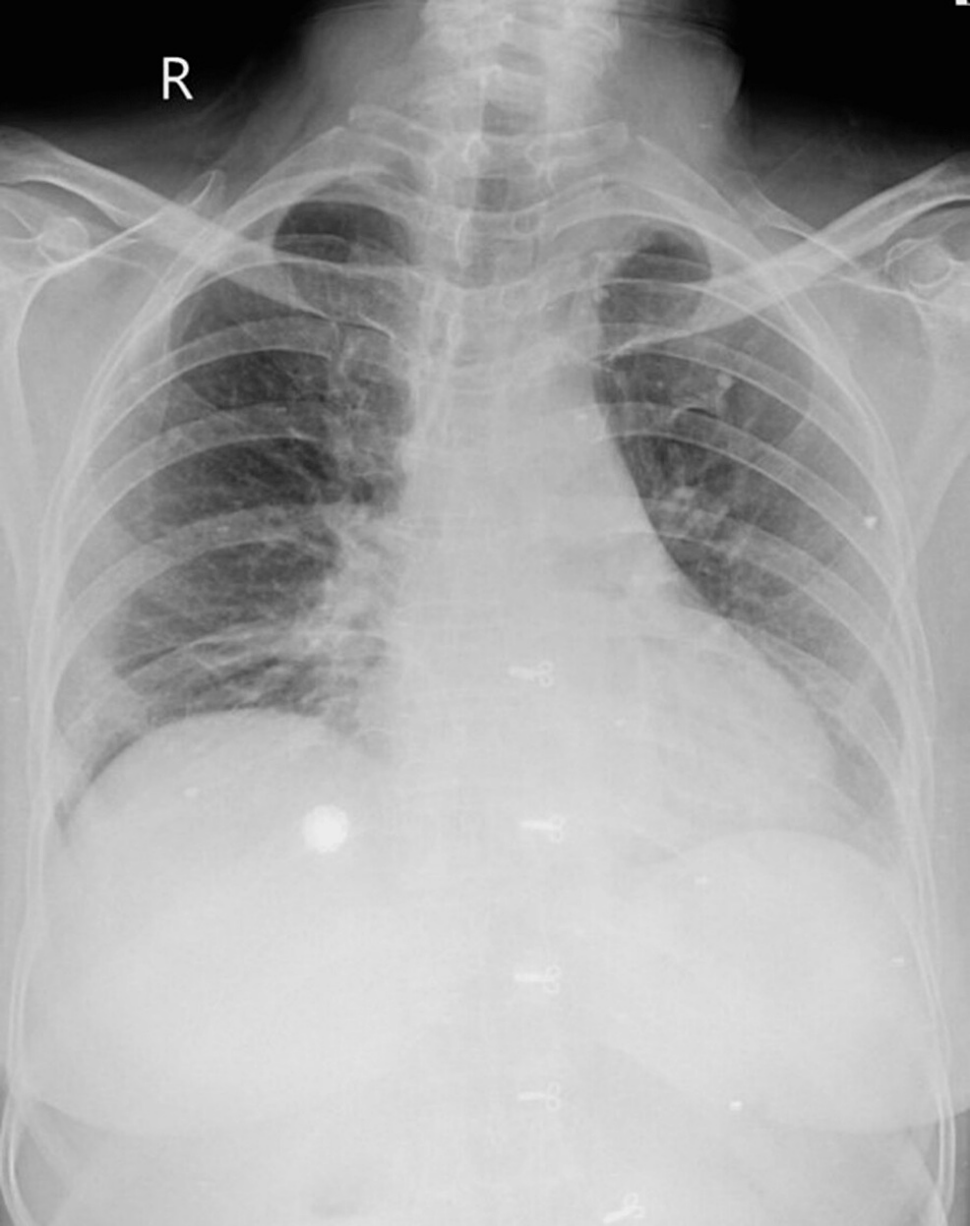
Chest X-ray of the patient on the day of discharge from the hospital.

**Figure 5:**
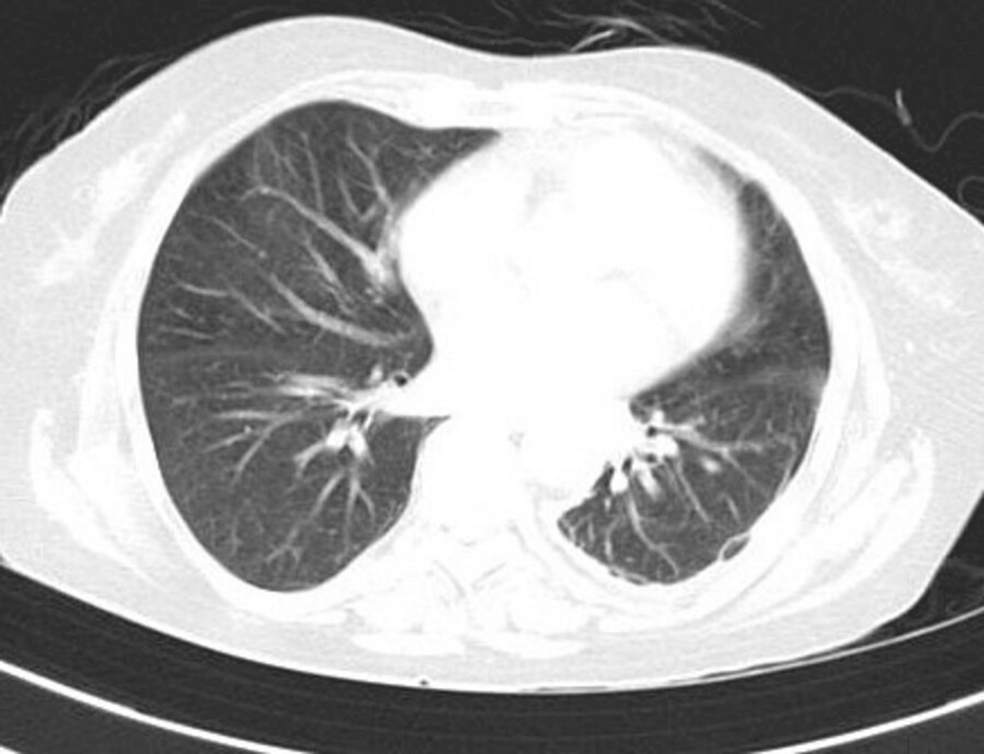
HRCT thorax of a patient after one month of discharge from the hospital. HCRT: high-resolution computed tomography.

The patient was subsequently admitted to the intensive care unit (ICU) for close monitoring and supportive care. The patient was put on invasive ventilation and started on intravenous glucocorticoids and antibiotics. Over the next few days, she slowly improved with continued treatment (Figure 3). She was eventually weaned off oxygen therapy and discharged home with a follow-up (Figure 4). One month after discharge, the patient presented for pulmonary re-evaluation and follow-up (Figure 5).

 

## Discussion

The diagnosis of dimethanol- and glutaraldehyde-induced chemical pneumonitis is based on a combination of clinical presentation, medical history, and diagnostic tests. Symptoms may range from mild coughing and chest discomfort to severe respiratory distress and hypoxemia. Chest imaging, such as an X-ray or computed tomography [CT] scan, may show diffuse bilateral infiltrates ground-glass opacities. Laboratory tests, such as arterial blood gas analysis or biomarker measurements, may help assess the severity and prognosis of the disease. The proposed theory of chemical pneumonitis may be acute lung injury due to inhalation or aspiration of irritant substances like gases, vapors, fumes, or particulates. In this case, reactive pulmonary edema or chemical pneumonitis may be due to dimethanol and glutaraldehyde fumigation. It is known to be toxic to the respiratory system, especially when exposed to high concentrations or for prolonged periods. Case reports are available highlighting the inflammatory response from chemical irritation that leads to temperature elevation within hours of exposure.

Treatment of dimethanol- and glutaraldehyde-induced chemical pneumonitis is mainly supportive and aimed at relieving symptoms and improving oxygenation. Oxygen therapy, bronchodilators, corticosteroids, and even mechanical ventilation may be necessary depending on the severity of lung injury [6]. Early recognition and prompt intervention are critical to reducing morbidity and mortality.

There are case reports of diffused alveolar damage due to drugs like acenocoumarol [7]. Interstitial pneumonitis sometimes may be confused with chemical pneumonitis which can happen after viral fever [8]. Employers and workers should be aware of the hazards of dimethanol and glutaraldehyde and follow regulatory requirements and guidelines. Health professionals should also educate patients and the public about the risks of chemical exposure and ways to protect themselves.

Each year, several people suffer ill health due to work-related hazards. Prevention of dimethanol and glutaraldehyde-induced chemical pneumonitis involves minimizing exposure to the substance through proper engineering controls, personal protective equipment, and safe work practices. This can be achieved by providing adequate staff training and using signs to highlight risks. Additionally, improved safety equipment, such as guards or additional personal protective equipment including goggles, safety boots, or high-visibility clothing, should be implemented. Replacing hazardous chemicals with less harmful alternatives is also essential.

Basic training should be given to all concerned staff, including those with limited practical experience. This training may involve observing fumigation from a place of safety (outside the risk area) or participating in the preparation for fumigation under the guidance of a qualified person.

## Conclusions

The dangers of exposure to dimethanol and glutaraldehyde from fumigation products must be thoroughly understood. The case highlighted the importance of appropriate training, safety precautions, and caution when handling hazardous compounds. It also emphasized the potential hazards that could arise from a single exposure. Fortunately, most of these poisonings can be avoided by taking necessary precautions.

It is crucial to carefully read and understand product labels, seek help when necessary, and use common sense when handling chemicals to avoid the risks associated with exposure. By prioritizing safety and taking necessary steps, we can minimize the possibility of unexpected results and promote a safe and healthy atmosphere for everyone. We believe that emergency physicians need ongoing training to effectively diagnose and manage patients who have been exposed to these substances.
